# P-2044. Patterns of Anaerobic Antibiotic Use for Community-Acquired Pneumonia

**DOI:** 10.1093/ofid/ofaf695.2208

**Published:** 2026-01-11

**Authors:** Rachel Alter, Regine Cherazard, Courtney Kluger, Jon Javor, Jason Pinksy, Scott Landman, Janette Hernandez-Torres, Miriam A Smith

**Affiliations:** LIJ Forest Hills Hospital, Forest Hills, NY; LIJ Forest Hills Hospital, Forest Hills, NY; LIJ Forest Hills Hospital, Forest Hills, NY; Department of Biostatistics/Feinstein Institute for Medical Research, Manhasset, New York; LIJ Forest Hills Hospital, Forest Hills, NY; LIJ Forest Hills Hospital, Forest Hills, NY; LIJ Forest Hills Hospital, Forest Hills, NY; Donald and Barbara Zucker School of Medicine at Hofstra/Northwell, Manhasset, New York

## Abstract

**Background:**

The 2019 American Thoracic Society (ATS) and Infectious Diseases Society of America (IDSA) guidelines for community-acquired pneumonia (CAP) in the absence of lung abscess or empyema do not recommend focused anaerobic antibiotic coverage. Inappropriate anaerobic coverage may lead to adverse effects and antimicrobial resistance. We assessed current prescribing practices for lower respiratory tract infections in the emergency department of a 312-bed academic community hospital that serves a diverse population in the New York City metropolitan area.

**Methods:**

We conducted a retrospective review of 120 randomly selected adult patients admitted with CAP between January 1 and December 31, 2023. Antibiotic regimens were evaluated for concordance with ATS/IDSA guidelines. Associations between antibiotic choice, patient characteristics, and clinical severity were examined. All conclusions are drawn at the p< 0.05 level, with analysis done in SAS v 9.4. Methods used were medians and interquartile ranges for summarization, and Wilcoxon Rank Sum Test for comparison of numeric outcomes. Frequency and percent were used for summarization, and Chi-Square/Fisher's Exact tests for comparison of categorical outcomes.

**Results:**

120 patients were analyzed (61 male; average age 72). The most common comorbid conditions were hypertension (52), diabetes (22), obstructive lung disease (21), and central nervous system disorders (16). Patients with sepsis were more likely to receive guideline-concordant therapy (p=0.017). A statistically significant association was demonstrated for patients with higher median white blood cell counts on admission and treatment with focused anaerobic antibiotics (WBC 10.30 versus 14.31, p=0.015). Piperacillin-tazobactam was more frequently prescribed in patients with hypertension (p= 0.03), diabetes (p= 0.02 and sepsis p= 0.02), regardless of indication.

**Conclusion:**

Clinical severity and comorbidities may influence inappropriate antibiotic selection, reflecting persistent misconceptions about the need for extended anaerobic coverage. Interventions to reinforce ATS/IDSA guideline adherence, including provider education and feedback on prescribing patterns, are warranted.

**Disclosures:**

All Authors: No reported disclosures

